# The effect of medication use on breastfeeding continuation: a systematic review with narrative synthesis

**DOI:** 10.1186/s13006-025-00756-y

**Published:** 2025-08-04

**Authors:** Rachel Pilgrim, Mo Kwok, Anthony May, Sarah Chapman, Matthew D. Jones

**Affiliations:** 1 Department of Life Sciences, University of Bath, Bath, UK; 2 Practice Plus Group Hospital, Shepton Mallet, UK; 3 Chelsea and Westminster Hospital NHS Foundation Trust, London, UK; 4 University Hospitals Plymouth NHS Trust, Plymouth, UK; 5 Institute of Pharmaceutical Science, King’s College London, London, UK

**Keywords:** Breastfeeding, Medication, Chronic, Barrier, Lactation, Drugs, Discontinuation, Medicines

## Abstract

**Background:**

Medication-related breastfeeding discontinuation occurs when women stop breastfeeding due to medication. While many medicines are safe during breastfeeding, women needing medication are less likely to continue. This disconnect may reflect avoidable barriers and missed opportunities for support. This review aimed to determine the proportion of postpartum women needing medication who discontinue breastfeeding, and to identify implicated medications, influencing factors, and risk factors.

**Methods:**

A systematic search of Embase, PubMed, Cochrane Library, PsycINFO, Scopus, and CINAHL was conducted from January 2004 to November 2024. Forward and backward citation searches were performed. Studies from high-income countries with self-reported breastfeeding discontinuation were included. Combination feeding and expressed breastmilk use were permitted. Exclusions included unpublished studies, non-English articles, case studies, HIV-related studies, alternative or illicit medicine use, and women who never initiated breastfeeding. Risk of bias was assessed using validated tools. Narrative synthesis was used to summarize findings.

**Results:**

Twenty studies (nine prospective cohort, five retrospective surveys, four qualitative, one randomised controlled trial and one cross-sectional mixed-methods study) were included. Discontinuation rates ranged from 2 to 18% (*n* = 293) in general populations and 2–58% (*n* = 1077) in women with chronic or severe acute conditions. Fourteen studies identified 29 medicines involved; all except lithium had post-marketing data indicating safety. Nine studies explored influencing factors. Healthcare professionals were described as encouraging discontinuation in three studies and reducing it in three studies. Other influencing factors were sparsely explored. One study identified risk factors, including lower education, Caesarean section, chronic conditions, employment at six months postpartum, less breastfeeding experience, and pre-pregnancy smoking (*p* < 0.05). The evidence base was limited by methodological heterogeneity, high bias risk, and low population diversity.

**Conclusions:**

Many instances of medication-related breastfeeding discontinuation may be avoidable, given the safety profiles of most implicated medicines. Inconsistent healthcare advice and systemic barriers likely contribute to unnecessary cessation. Further research should explore sociocultural, psychological, and systemic influences on decision-making, particularly among underrepresented groups, to inform equitable, effective interventions that support both breastfeeding and maternal health.

**Supplementary Information:**

The online version contains supplementary material available at 10.1186/s13006-025-00756-y.

## Background

Breastfeeding is a critical public health intervention. It is associated with substantial short- and long-term health benefits for both infants and mothers. For infants, it reduces the risk of acute infections, sudden infant death syndrome, and chronic conditions such as obesity and type 2 diabetes. For mothers, breastfeeding is linked to a reduced risk of breast and ovarian cancer, type 2 diabetes, and cardiovascular disease [1]. It also supports maternal–infant bonding and may improve mental health outcomes [2]. Consequently, any preventable barrier to breastfeeding, such as lack of clarity around medication use, carries implications not only for individual families but also for health systems and societies more broadly [3, 4]. World Health Organization guidance recommends exclusive breastfeeding for the first six months of an infant’s life. Breastfeeding is then recommended alongside solid food until at least two years of age [5]. This is also reflected in national guidance from high income countries such as the UK, USA and Canada [6–9]. In the UK, breastfeeding rates at 6–8 weeks of age are reported annually. Data for 2023 shows that an average of 49% of women are breastfeeding at this time point [10]. Current data on exclusive breastfeeding at six months is unavailable. The UK Infant Feeding Survey conducted almost 15 years ago found that only 1% of babies were being exclusively breastfed at six months, and only 34% and 0.5% of babies were receiving any quantity of breastmilk at six and 12 months, respectively [11]. This represents one of the lowest rates in Europe [12]. Exclusive breastfeeding at 6 months is reported in 27.2% of infants in the USA, 38% in Canada, and 37.5% in Australia [13–15].

Factors behind discontinuation of breastfeeding have previously been explored in a survey of 500 mothers in the UK [16]. This revealed some of the perceived barriers to breastfeeding, which included embarrassment, worries about pain, concerns about the child receiving too much or not enough milk, and feeling they are “tied down and stopped from doing what they want.” 71% of participants agreed with the statement “it could prevent me from taking medication”. A recent scoping review also found maternal medication to be an important factor in breastfeeding discontinuation in studies conducted in Saudi Arabia, Taiwan, Italy and the USA [17].

The maternal use of medication whilst breastfeeding does require caution, as some medicines may affect the breastfed infant. However, disproportionate concern or lack of nuanced guidance can lead to unnecessary breastfeeding cessation. This may deprive both mother and infant of the well-established short- and long-term health benefits of breastfeeding, including protection against infections in infants and chronic disease risk reduction in mothers. The extent to which a drug can affect an infant depends on the amount of drug or active metabolite that is transferred into breastmilk as well as pharmacokinetic factors in the infant. Infants are most likely to be exposed to medications taken by the mother through her breastmilk in the first few days following delivery. At this stage, intercellular gaps in the milk ducts are wide open, allowing for large molecules such as antibodies to pass through. Once these gaps have closed, drugs may still pass into milk, but this is dependent on that drug’s ability to cross cell membranes. For many drugs, the amount transferred into breastmilk is insufficient to produce any harmful effect on the infant [18–20].

Many medicines are not licensed for use during breastfeeding due to the frequent exclusion of breastfeeding women from clinical trials. This exclusion is driven by ethical, legal, and logistical concerns, such as the potential risk of drug transmission to the infant via breastmilk, challenges in assessing benefit-risk for the mother-infant dyad, and perceived legal liability. Regulatory uncertainty, inconsistent application of ethical guidelines, and complex approval processes further discourage research in this population. These barriers result in a lack of robust safety data, leading to conservative licensing decisions and ongoing uncertainty regarding medication use during breastfeeding [21]. However, once a medication becomes widely used, the incremental accumulation of clinical experience and publication of research enables an assessment of compatibility. Assessment of pharmacokinetic data can also be useful for predicting the ability of a drug to reach the infant. However, advice in product licenses (which is reflected in official formularies such as the British National Formulary in the UK) is usually limited. The UK’s National Institute for Health and Care Excellence (NICE) advises that such guidance is “used as a guide only, as it does not contain quantitative data on which to base individual decisions” and advises that prescribers consult supplementary sources [22]. Several resources provide such information about drug safety in breastfeeding, including the UK Drugs in Lactation Advisory Service [23], LactMed^®^ [24], and the Drugs in Breastmilk Service factsheets [25]. NICE also states that in most cases, it is possible to identify a suitable medication that is safe for use in breastfeeding, and continued breastfeeding should be encouraged [22]. There is therefore substantial support available for both breastfeeding individuals and healthcare professionals who require more information about the safety of a medication in breastfeeding. However, it is possible that many women and healthcare professionals are unaware of these services [26]. This highlights a critical need for improved multidisciplinary education and support for healthcare professionals including prescribers, pharmacists, and midwives, to better equip them to counsel breastfeeding women. Addressing gaps in knowledge and confidence among providers is a modifiable barrier with the potential to significantly impact breastfeeding continuation rates.

Healthcare professionals may be contributing to the problem of early breastfeeding discontinuation due to medication. An evaluation of the Drugs in Breastmilk service revealed that 21% (*n* = 57) of mothers who contacted the service did so after being advised to cease breastfeeding by their initial source of guidance. Upon assessment by the service, 98% (*n* = 56) were found to have received inaccurate advice and could safely continue breastfeeding. Most women found the service via Facebook or breastfeeding groups, with less than 6% being referred by conventional healthcare providers [26]. The extent to which over-cautious medical advice or personal misconceptions have led to breastfeeding discontinuation or non-initiation among UK women who are unaware of the Drugs in Breastmilk service remains unclear.

Obtaining a deeper understanding of this issue would be invaluable. If it is identified that women are regularly choosing to discontinue breastfeeding due to a medication that is known to be compatible with breastfeeding, it is important to understand why this is happening and to identify risk factors associated with this decision. This would allow for the design of targeted interventions, which could ultimately contribute to improving breastfeeding rates, allowing women and their children to have the best chance of realising the health benefits associated with breastfeeding.

This review aims to inform future policy, practice, and intervention development by synthesizing current evidence on how medication use impacts breastfeeding decisions. By identifying modifiable risk factors and key points of influence, this review will support the design of targeted strategies to reduce unnecessary breastfeeding cessation and promote equitable access to breastfeeding-compatible healthcare. The primary objective is to determine the incidence of breastfeeding discontinuation among postpartum women who require medication. Secondary objectives include identifying the medicines associated with these decisions, examining factors influencing women in their choice to continue or discontinue breastfeeding while on medication, and exploring potential associations between a woman’s socio-economic, health, geographic, or demographic background and her likelihood of continuing breastfeeding whilst using medication.

## Methods

The Preferred Reporting Items for Systematic Reviews and Meta-Analyses (PRISMA) statement was used throughout the systematic review [27]. The protocol was published in advance (PROSPERO CRD42021285611).

### Eligibility criteria

Primary research published within 20 years of the date of the final search was eligible for inclusion. This timespan was chosen to ensure a comprehensive review, given the expected limited volume of relevant studies, and to reflect changes in information access following the widespread adoption of the internet, which likely influenced both maternal behaviour and clinical guidance.

All study designs were eligible for inclusion aside from case studies and case series. All studies concerning the delivery of a woman’s own breastmilk to an infant, whether this is in the form of exclusive breastfeeding, combination feeding involving the administration of both formula and breastmilk, or the administration of expressed breastmilk via a bottle were permitted. This review solely considered women who had successfully initiated breastfeeding who then presented with the need to take medication whilst breastfeeding. Medication-related breastfeeding discontinuation is defined as the permanent cessation of breastfeeding, where medication is reported as one of the contributing reasons. Temporary cessation followed by a return to breastfeeding is not classified as medication-related breastfeeding discontinuation. Papers concerning women who never initiate breastfeeding due to medication were excluded.

Studies focusing on illicit medication use, complementary or alternative medicines, galactagogues being used for the indication of increasing milk supply, or drugs being used for the indication of suppressing lactation were excluded. Aside from this, there were no restrictions on medication indication, pharmacological class, route of administration or legal status (e.g. prescribed or purchased over the counter). Unpublished studies, conference proceedings and abstracts were excluded. Studies published in languages other than English were excluded. Studies that explicitly stated they involved women with HIV were excluded due to potential confounding by concerns around viral transmission. However, studies that did not specify HIV status were included.

Only studies carried out in high-income countries were included, as defined by current World Bank classification [28]. If a study contained data from both high- and low-income countries, it was included if the data for high-income countries could be clearly extracted. This was to allow more valid comparison between studies from multiple countries; it is known that breastfeeding rates differ between high- and low-income countries and influences on breastfeeding decisions are likely to be very different [1].

### Search strategy

The search strategy was developed with the help of a health service librarian. Search terms were developed using synonyms of the following concepts: breastfeeding, medication and discontinuation. The full search strategy can be found in Additional File 1- Search Strategy. Embase (Elsevier), MEDLINE (PubMed), the Cochrane Library (including the Cochrane Central Register of Controlled Trials) (Wiley), PsycINFO (APA PsycNET), Scopus (Elsevier) and CINAHL (EBSCOhost) were searched. Forward and backward citation searches were carried out for all included studies. Databases were searched from 2004 through to the final date of search in October 2024.

### Study selection

Following identification and removal of duplicate studies, the lead author (RP) screened all abstracts against the eligibility criteria. 11% of abstracts were randomly selected for screening by a second author (MK and AM). Full texts were retrieved for all studies that initially met the eligibility criteria and were screened by the lead author (RP). 10% of the full texts were randomly selected for independent screening by a second author (MK or AM). Disagreements were resolved by discussion. Inter-rater reliability of both screening stages was assessed with Cohen’s kappa (SPSS v28, IBM).

### Assessment of methodological quality

All quantitative studies were assessed for bias. Retrospective and prospective cohort studies were assessed for methodological quality using the Newcastle-Ottawa Scale [29], which was modified by the authors of this review to ensure it was relevant for single cohort studies. For cross-sectional studies, an adapted Newcastle-Ottawa Scale was used [30]. This tool was further modified by the authors of this review to ensure it assessed similar areas as the original Newcastle-Ottawa Scale for cohort studies. This included removal of questions regarding clarity of aims and sample size, and inserting the option to select “not applicable” for questions regarding comparability between groups to allow for studies with single outcome groups. The Jadad Scale was used to assess bias in the randomised controlled trial [31]. Bias assessment was completed by a single author (RP). All bias assessment tools can be found in Additional File 4- Bias Assessment. 20% of the quality assessments were double checked by a second reviewer (MK). Studies were not excluded based on their quality. Risk of bias due to missing results (reporting biases) was assessed qualitatively. During the data extraction process, studies with incomplete or missing data were flagged.

### Data extraction

Data was extracted by the lead author (RP). Data extracted included year of publication, year of data collection, study design, study aims, country of recruitment, sample size, duration of follow-up, inclusion and exclusion criteria, method of recruitment, number of participants, missing results, data on infant ages and type of feeding, proportion of breastfeeding individuals who need to start a medication that choose to discontinue breastfeeding as a result, medicines contributing to this decision, factors influencing this decision, and any reported associations between a woman’s socio-economic, health, geographic or demographic background and her likelihood of continuing breastfeeding while using medication.

### Data synthesis

Due to wide variation in disease type/severity, medication risk profile, and study designs, meaningful meta-analysis of quantitative outcomes was not possible. High heterogeneity was observed in study populations, outcome definitions, and reporting methods, preventing direct statistical pooling. Instead, narrative synthesis was applied to data related to each outcome, following the synthesis without meta-analysis (SWiM) in systematic reviews reporting guidelines [32].

For quantitative studies, results were grouped by outcome type to allow for comparison across studies. Studies reporting the proportion of women discontinuing breastfeeding due to medication use were summarized in tables. To account for differences in medication use and breastfeeding discontinuation, populations were categorized into two groups: general postpartum women and those with chronic or severe acute conditions. This classification was not part of the original protocol but was introduced after reviewing the literature as it allowed for more meaningful comparisons. Medicines cited as contributing to medication-related breastfeeding discontinuation were compiled, with safety classifications noted where available using Hale’s lactation risk categorisation [18]. Studies exploring reasons for discontinuation were analysed thematically, identifying common themes such as healthcare advice and perceived risks. Finally, studies reporting statistical associations between socio-demographic or clinical factors and medication-related breastfeeding discontinuation were summarized, with effect measures such as odds ratios and *p*-values extracted where available. To aid interpretation, study characteristics and key results were presented in tables. Figures were used where appropriate to illustrate key trends.

Heterogeneity was assessed qualitatively by comparing study designs, populations, and outcome definitions. No formal statistical heterogeneity assessment (e.g., I²) was conducted due to the absence of meta-analysis. Certainty or confidence in the body of evidence for each outcome was not formally assessed in this review.

Where necessary, data conversions were performed to ensure comparability between studies. If discontinuation rates were reported inconsistently (e.g., percentages vs. raw numbers), these were standardized as proportions. No formal imputation methods were used. No formal sensitivity analyses were conducted due to the narrative nature of the synthesis and the heterogeneity of included studies.

### Certainty of evidence

The certainty of evidence was assessed using the Grading of Recommendations Assessment, Development and Evaluation (GRADE) approach [33]. The following domains were considered: risk of bias, inconsistency, indirectness, imprecision, and publication bias. We rated down from an initial level of “low” due to the observational design of included studies. GRADE assessments were summarised in a Summary of Findings table.

## Results

Searches identified 4808 unique articles after duplicate removal (see Additional File 2- PRISMA Flow Diagram). The full text of 292 articles was reviewed from database searches, along with an additional 94 articles from citation searches. This resulted in 20 studies identified for inclusion (see Table 1 for study details; a more comprehensive version can be found in Additional File 3– Table of Study Characteristics). Cohen’s kappa during title and abstract screening was 0.87. There was full agreement between raters during full text screening.

**Table 1:** Summary of included studies

Study and study type	Setting	Participants details	Sample size	Data collection
De Waard et al. [34] Prospective cohort	Netherlands Single centre in Amsterdam	All women giving birth in obstetrics ward, or under midwife-led care at three midwifery offices.	292 women	Structured interview 2 to 5 weeks postpartum. Data from medical chart. Postal questionnaire within 4 months.
Gilad et al. [35] Prospective cohort	Israel Calls to teratology information service helpline	All breastfeeding women asking about olanzapine	37 women requiring olanzapine 51 women using paracetamol as a control group	Telephone questionnaire 1–2 years after initial contact
Gilad et al. [36] Prospective cohort	Israel Calls to teratology information service helpline	Breastfeeding women asking about methylergonovine	38 women requiring methylergonovine 58 women using amoxicillin as a control group	Telephone questionnaire 1–3 years after initial contact
Aigner et al. [37] Retrospective survey	Germany Three hospital neurology clinics	Women with multiple sclerosis who gave birth in previous 3 years	62 women giving birth to 64 children, of whom 55 were breastfed	Single questionnaire
Baker et al. [38] Retrospective mixed-methods survey	England and Wales Acute psychiatric settings nationwide	Women with psychiatric disorder requiring acute care during the first year after childbirth	218 women, of whom 144 reported initiating breastfeeding.	Questionnaire 1-month post discharge
Ince-Askan et al. [39] Prospective cohort	Netherlands Nationwide	Women with rheumatoid arthritis who wished to conceive or were already pregnant	249 pregnancies from 216 women	Questionnaire at 6, 12, and 26 weeks postpartum
Kemper et al. [40] Prospective cohort	Netherlands Single centre	Women with rheumatoid arthritis who had given birth	171 pregnancies; 120 women reported initiating breastfeeding	Face-to-face questionnaire during each trimester and postpartum at 4–6, 12 and 26 weeks.
Klevmoen et al. [41] Retrospective survey	Netherlands and Norway Nationwide	Women with familial hypercholesterolaemia who had given birth to live children	102 women, of whom 78 report initiating breastfeeding	Single online questionnaire
Mills et al. [42] Retrospective cohort	USA Single centre	Women with autoimmune or inflammatory rheumatic diseases aged 18 to 50 who were currently pregnant or had previously had a successful pregnancy	151 pregnancies from 90 respondents, of which 82% were breastfed.	Single survey. Time point postpartum unclear.
Orefice et al. [43] Prospective cohort	Italy Single centre in Rome	Women with systemic lupus erythematosus, enrolled prenatally	57 pregnancies involving 43 women. In 41 of these pregnancies breastfeeding was initiated.	Questionnaire at several time points before/after birth. Reasons for breastfeeding discontinuation reported at 1 month only
Tandon et al. [44] Prospective cohort	Canada Single centre	Ambulatory adult women with inflammatory bowel disease presenting to a tertiary pre-conception and pregnancy clinic	74 women, of whom 70 initiated breastfeeding	Survey at 0, 3, 6 and 12 months postpartum.
Frayne et al. [45] Qualitative study	Australia Single clinic setting	Women attending an antenatal clinic for severe mental illness	12 women	Single interview 4–6 weeks postpartum
Hicks et al. [46] Cross-sectional mixed methods	USA Single opioid dependence treatment centre	Women over 18 who delivered a baby while in treatment	30 women	Single interview
Ikram et al. [47] Prospective cohort	USA Single clinic	Women with rheumatic disease	265 pregnant women; 222 initiated breastfeeding	Survey completed during pregnancy and 5–12 weeks after delivery
Lewallen et al. [48] Cross-sectional qualitative data	South-eastern USA Community or specialty women’s hospitals	Women who gave birth to a term neonate requiring routine care only, breastfeeding for the first time, intending to breastfeed for at least 8 weeks	379 women, of which 121 had ceased breastfeeding at time of data collection	Single telephone interview at 8 weeks postpartum
Standish et al. [49] Qualitative	USA Boston Medical Centre and other speciality clinics	Women with a diagnosis of opioid use disorder who were primary care takers of their infants	23 women, of whom 16 initiated breastfeeding	Single semi-structured interview
Tigka et al. [50] Prospective cohort	Greece Five tertiary maternity hospitals in one prefecture	Mothers who had given birth and were hospitalized in the postnatal ward	847 women	In-person structured interview conducted on third day postpartum. Follow-up telephone interview at 1, 3 and 6 months
Zingone et al. [51] Cross-sectional	Italy Single centre	Women aged 18–65 years with IBD diagnosis of at least 1 year	228 women with IBD, and 229 healthy controls. 61 IBD patients provided information on childbirth; 41 breastfed	Single questionnaire
Lewkowitz et al. [52] Randomised controlled trial	USA Single centre	Socioeconomically disadvantaged African American women aged 18–35 years with BMI 30–45 kg/m^2^ with established prenatal care	118 women; 59 assigned to each study arm	Randomly assigned to a home-based parenting support and child development educational intervention, with or without additional content on breastfeeding. Single telephone questionnaire, 6–12 months postpartum
Teich et al. [53] Qualitative study of women enrolled in separate randomised controlled trial	USA Two sites in New York	Women aged over 18 in the first or second trimester of pregnancy	67 women	Single interview at 6 months postpartum

BMI: body mass index; IBD: inflammatory bowel disease; USA: United States of America

### Methodological quality

The complete methodological quality assessment of quantitative studies can be found in Additional File 4- Bias Assessment. Inter-rater reliability, measured using Cohen’s kappa, was found to be 0.69. Using the Newcastle-Ottawa Scale for cohort studies, the quality scores for studies ranged from 17 to 83%, with a mean of 51%. Using the modified Newcastle-Ottawa scale for the cross-sectional studies, study quality ranged from 27 to 64%, with an average of 45%. For both cohort and cross-sectional studies, there were limitations due to the duration or time point of follow-up; many studies collected data before the 6-month postpartum time point.

The one randomised controlled trial was assessed to have high quality for the randomisation and dropouts domains, but lower quality in the blinding domain due to being single-blinded. However, given it was comparing types of support given to postpartum women, double-blinding would not have been possible.

### Study outcomes

#### Incidence of breastfeeding individuals requiring medication choosing to discontinue breastfeeding

Three included studies reported data on the proportion of breastfeeding women presenting with a need to start a medication, who discontinued breastfeeding as a result. Across these studies, the proportion of women needing medication who opted to discontinue breastfeeding ranged from 2 to 18%. All of these studies consisted of general populations of women (Table 2a).

**Table 2a:** Incidence of medication-related breastfeeding discontinuation in general populations of breastfeeding women

Study	Results
De Waard et al. [34]	2 of 109 (2%) women using medicines stopped breastfeeding because of the need to start medication.
Gilad et al. [35]	4 of 37 women (11%) did not breastfeed due to fear of olanzapine effects on the infant and 5 of 37 (14%) stopped due to initiation of another drug. 11 women stopped breastfeeding due to “medical advice”. No further explanation is provided, and whether this concerns medication. One woman also stopped breastfeeding due to fear. It is unclear if this is related to medication. In total, excluding those who discontinued due to medical advice and undefined fear, 9 women (24%) in the olanzapine group report medication-related breastfeeding discontinuation. No women (0%) from the control group discontinued breastfeeding due to paracetamol use
Gilad et al. [36]	7 of 38 women (18%) stopped breastfeeding because of concerns regarding methylergonovine 3 of 58 mothers (5%) from the control group discontinued breastfeeding due to concerns about infant exposure.

**Table 2b:** Incidence of medication-related breastfeeding discontinuation in women with chronic or severe acute conditions

Study and study type	Clinical condition	Results
Aigner et al. [37]	Multiple sclerosis	16 of 55 (29%) babies were weaned due to starting immunotherapy. No elaboration on which immunotherapies this concerned
Baker et al. [38]	Acute psychiatric disorders	43 of 144 (30%) breastfeeding women report stopping breastfeeding earlier than they planned due to the concurrent use of medication.
Ikram et al. [47]	Rheumatic disease	8 of 222 women (4%) who breastfed report breastfeeding discontinuation due to concerns about medication. Note that a further 25 pregnant women (9%) report never initiating breastfeeding due to medication concerns.
Ince-Askan et al. [39]	Rheumatoid arthritis	Of the women reporting breastfeeding discontinuation before 26 weeks, in 129 of 223 pregnancies (58%) the reason for discontinuation of breastfeeding was restart of medication
Kemper et al. [40]	Rheumatoid arthritis	15 of 74 women (20%) who provided information on breastfeeding discontinuation stopped breastfeeding due to medication before 26 weeks postpartum. Unclear if these women are just those exclusively breastfeeding, or both exclusive and partial.
Klevmoen et al. [41]	Familial hypercholesterolaemia	17 of 78 (22%) breastfeeding women cited restarting statin treatment as the reason for breastfeeding discontinuation.
Mills et al. [42]	Rheumatic disease	Fourteen women with active disease explained that they stopped breastfeeding because they needed to start a medication for their condition that was thought to be contraindicated. The paper reports this as 12% of the 90 respondents, however some of these respondents did not initiate breastfeeding at all.
Orefice et al. [43]	Systemic lupus erythematosus	One woman out of 41 (2%) reported breastfeeding discontinuation at one month due to medication initiation.
Tandon et al. [44]	Inflammatory bowel disease	Four women out of 70 (6%) reported that the primary factor for discontinuing breastfeeding was due to concerns about new or existing medication exposure to the baby.

A further nine studies focused on populations of women with specific chronic or severe acute conditions that are ordinarily managed with medicines, therefore it is reasonable to assume that most participants had a need for medication. In the included studies, among the population of women with chronic diseases who had initiated breastfeeding, the proportion of women who then opted to discontinue breastfeeding due to a need for medication ranged from 2 to 58% (Table 2b).

Substantial variability in study design, medication type, follow-up period and healthcare settings likely contributed to this result. For example, within the studies focusing on women with chronic diseases, a Dutch cohort study of women with rheumatoid arthritis found 58% of participants had discontinued breastfeeding due to a medication by 26 weeks postpartum [39]. Conversely, an Italian cohort study of women with systemic lupus erythematosus found that only 2% of women reported breastfeeding discontinuation due to a medicine, although this information was collected at 4-weeks postpartum only [43].

#### Medicines contributing to women discontinuing breastfeeding

**Fig. 1:**
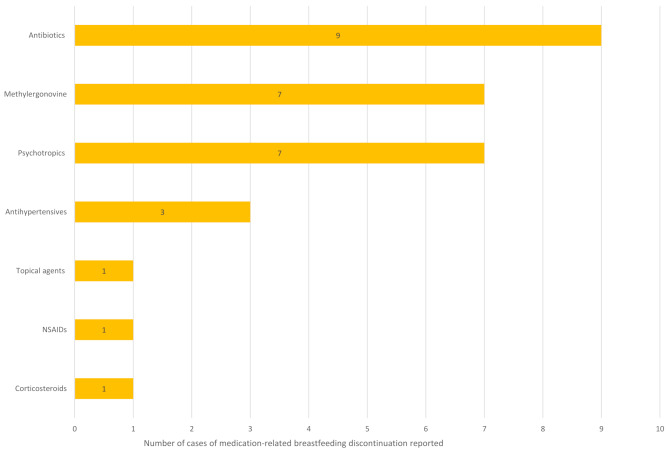
A summary of cases of medication-related breastfeeding discontinuation in general populations according to drug class. Note in some cases more than one drug may be involved in an individual case; all drugs are included separately. Only studies reporting quantitative data on medications involved in medication-related breastfeeding discontinuation are included (*n* = 6)

**Fig. 2:**
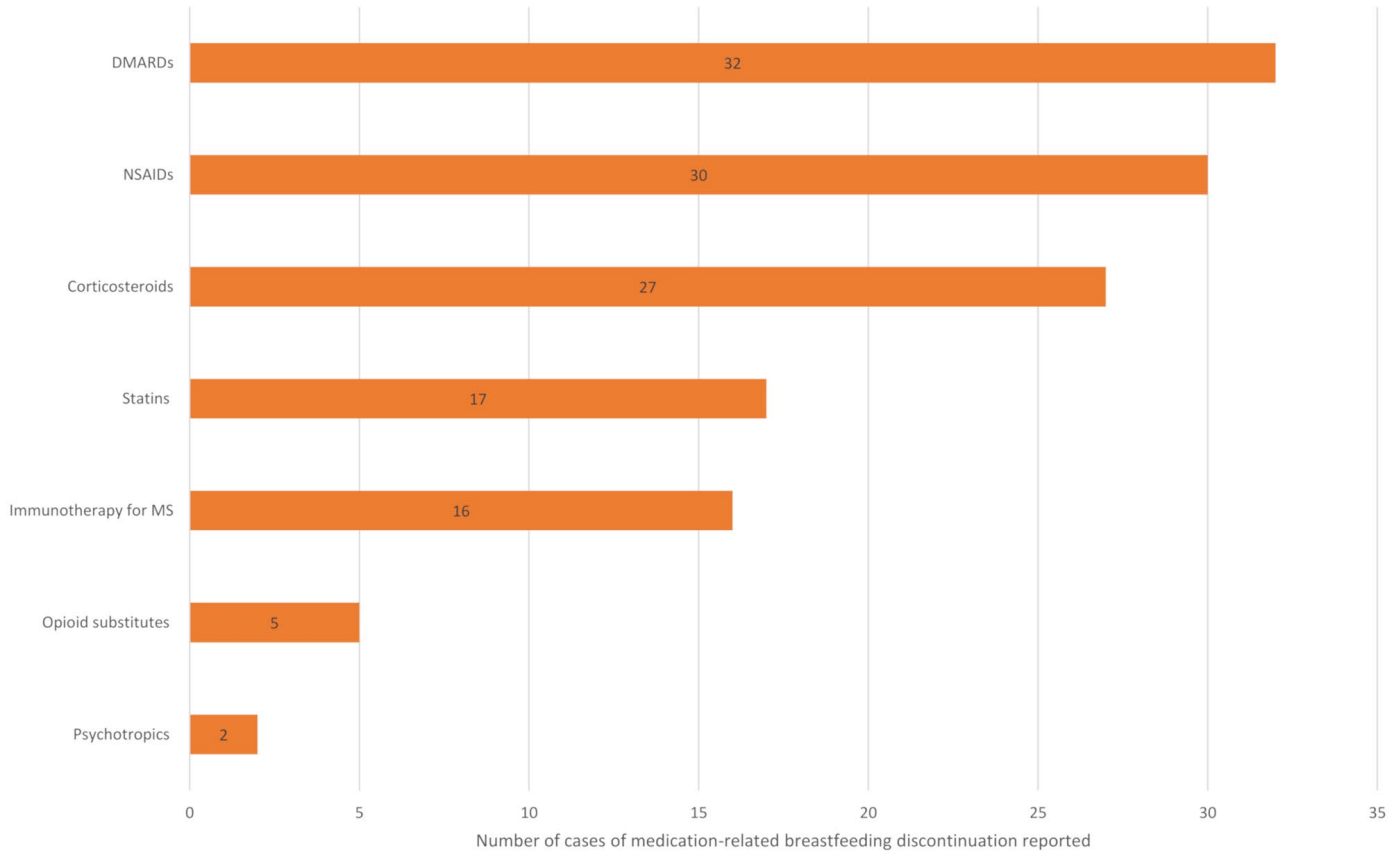
A summary of medication-related breastfeeding discontinuation in chronic disease populations according to drug class. Note in some cases more than one drug may be involved in an individual case; all drugs are included separately. Only studies reporting quantitative data on medications involved in medication-related breastfeeding discontinuation are included (*n* = 6)

Fifteen studies provided data on the specific medications involved in medication-related breastfeeding discontinuation (see Supplementary Table 1 in Additional File 5–- Results Tables). By far the most widely studied area is disease-modifying anti-rheumatic drugs and other drugs associated with the management of rheumatic conditions, such as steroids and non-steroidal anti-inflammatory drugs (NSAIDs), with four studies reporting on these [40, 42, 43, 47]. One study provides data focusing on immunotherapy in multiple sclerosis [34] and one paper provides data on statin usage in familial hypercholesterolaemia [41]. A summary of the cases of medication-related breastfeeding discontinuation according to drug class can be found in Figs. 1 and 2.

**Fig. 3:**
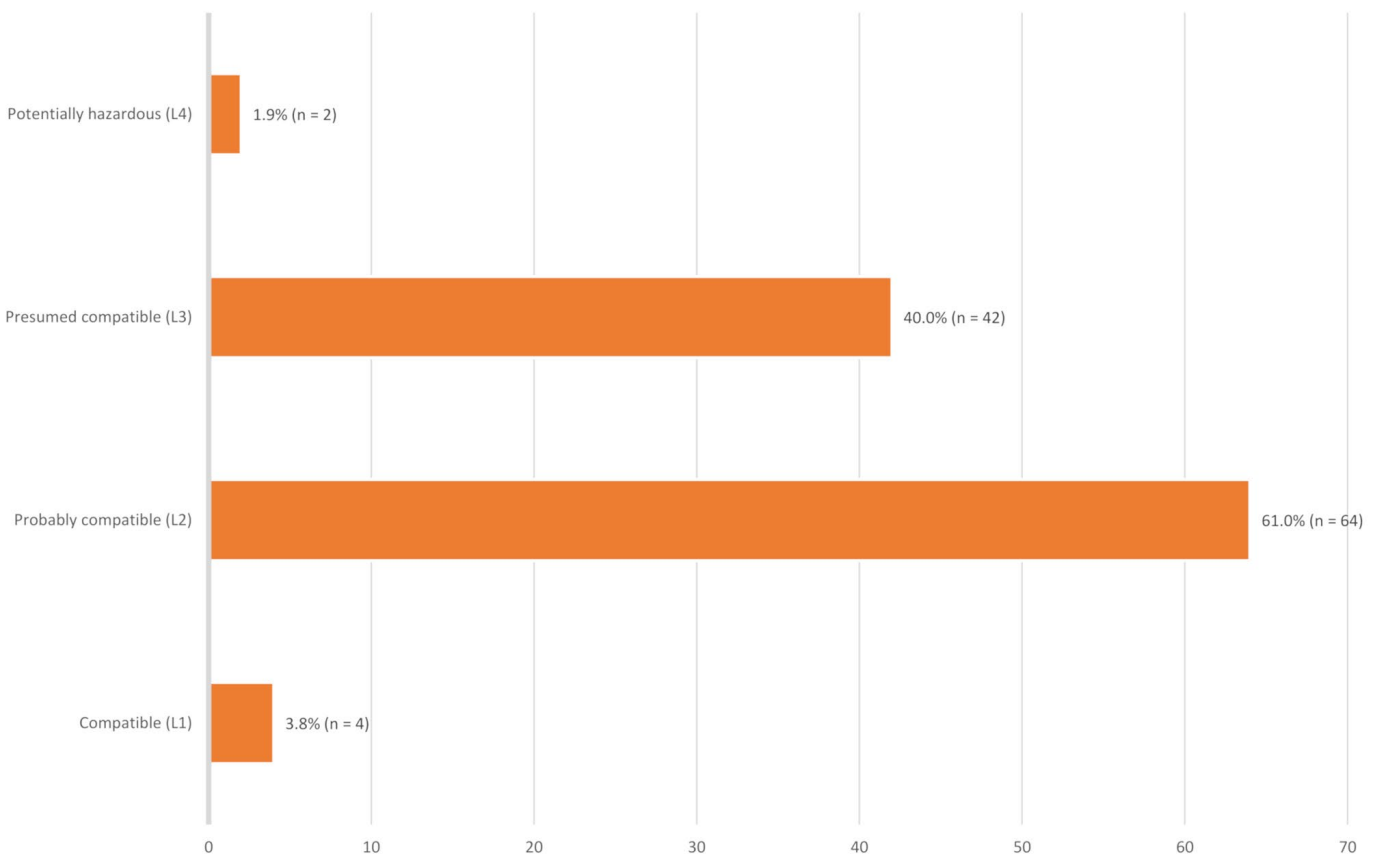
Summary of the risk categories of drugs involved in medication-related breastfeeding discontinuation, based on the number of cases identified. Note that some drugs were linked to multiple cases, meaning the total number of cases is higher than the number of distinct drugs. In some cases more than one drug may be involved in an individual case; all drugs are included separately. This only includes studies (*n* = 11) that provide quantitative data on individual drugs, rather than groups of drugs. No studies reported medication-related breastfeeding discontinuation due to a drug classified as L5 (hazardous)

Most of the individual drugs implicated in medication-related breastfeeding discontinuation are considered to be safe during breastfeeding, or at least can be used with a degree of caution according to Hale’s lactation risk categorisation [18]. A summary of the risk categories of medicines involved in medication-related breastfeeding discontinuation can be found in Fig. 3.

#### Factors influencing a woman in choosing to discontinue breastfeeding due to concurrent use of medication

Nine studies provided data on factors influencing women’s decisions to discontinue breastfeeding due to medication (one mixed-methods questionnaire, one randomised controlled trial, two qualitative interview studies and five prospective cohort studies). Full results can be found in Supplementary Table 2 (see Additional File 5– Results Tables). In six studies, healthcare professional advice was found to be an influencing factor, whether positively [40, 52, 53] or negatively [38, 43, 50]. Three papers provide evidence of healthcare professional advice causing women to discontinue breastfeeding early due to medication, with a range of healthcare professionals involved including midwives, health visitors, general practitioners, paediatricians and other physicians [38, 43, 50]. One paper calculated that a physician recommendation to discontinue breastfeeding due to medication was significantly correlated with medication-related breastfeeding discontinuation (*p* < 0.05) [50]. This study also found that of five cases of women discarding breastmilk or reducing the frequency of feeding on the advice of a healthcare professional, all five resulted in early discontinuation of breastfeeding due to a reduction in milk supply. Conversely, structured support from appropriately trained healthcare professionals, such as lactation consultants [53] and targeted interventions [52], helped women navigate medication use while breastfeeding, reducing unnecessary discontinuation.

In four studies, a woman’s personal concerns were reported as a significant factor [35, 36, 49, 50]. In most studies, an in-depth assessment of these personal concerns was not made. Qualitative work in a population of women using opioid substitution therapy identified themes related to the decision to discontinue breastfeeding due to opioid substitution therapy. These included scepticism and concerns about medication exposure, perceived lack of control over breastfeeding decisions, and beliefs about the impact of breastfeeding on infant withdrawal [49].

#### Associations between women’s personal characteristics, and her likelihood of continuing breastfeeding whilst using medication

Two studies provided data on risk factors associated with medication-related breastfeeding discontinuation (see Supplementary Table in Additional File 5– Results Tables). This was most extensively studied in a single paper, which found lower educational level, birth via Caesarean section, the use of medication for a chronic condition, employment at 6 months postpartum, fewer days breastfeeding experience, and smoking pre-pregnancy were all associated with the decision to discontinue breastfeeding due to medication (*p* < 0.05) [50]. No association was found with maternal age, nationality, parity, or employment before pregnancy. Another study also found that those on a greater number of medications were more likely to discontinue breastfeeding after commencing olanzapine [35].

#### Summary of findings and certainty of evidence

All outcomes were rated very low certainty due to study limitations, inconsistency, and possible publication bias. See Table 3 for detail.

**Table 3:** Summary of findings and GRADE certainty of evidence for key outcomes

Outcome	Participants (number of studies)	Effect estimate	Certainty of evidence (GRADE)	Comments
Incidence of breastfeeding discontinuation due to medication (general population)	242 (3)	Proportion of breastfeeding individuals reporting medication-related discontinuation ranged from 2–18% across studies.	VERY LOW	Downgraded for serious risk of bias (concerns around sampling and outcome assessment), inconsistency (substantial variability in effect size across studies), indirectness (limited generalizability), small total sample size and possible publication bias
Incidence of breastfeeding discontinuation due to medication (individuals with chronic diseases)	997 (9)	Proportion of breastfeeding individuals reporting medication-related discontinuation ranged from 2–58% across studies.	VERY LOW	Downgraded for serious risk of bias (concerns around sampling and outcome assessment), inconsistency (substantial variability in effect size across studies), indirectness (limited generalizability), and possible publication bias
Types of medication commonly associated with medication-related breastfeeding discontinuation	2732 (15)	Wide range of drug classes implicated. DMARDs, NSAIDs, and steroids were most frequently reported. Of 112 total medication-related breastfeeding discontinuation cases, only 2 involved potentially hazardous (L4) drugs; the remaining 110 involved drugs classified as “compatible”, “probably compatible” or “presumed compatible” (L1–L3).	VERY LOW	Downgraded for serious risk of bias (sampling and outcome assessment), indirectness (limited generalizability) and possible publication bias
Factors influencing medication-related breastfeeding discontinuation	1715 (7)	Healthcare professional advice influenced decisions both positively and negatively. Three studies linked healthcare professional directly to medication-related breastfeeding discontinuation. One study found a significant correlation (*p* < 0.05) between healthcare professional advice and medication-related breastfeeding discontinuation. Support from lactation consultants reduced medication-related breastfeeding discontinuation. Women’s personal concerns (e.g. safety fears, loss of control) also contributed.	VERY LOW	Downgraded for serious risk of bias (sampling and outcome assessment), indirectness (limited generalizability) and possible publication bias
Sociodemographic and clinical predictors of medication-related breastfeeding discontinuation	935 (2)	One study identified lower education, Caesarean birth, chronic illness, employment at 6 months, shorter breastfeeding duration, and smoking as significant predictors (*p* < 0.05). No association with maternal age, parity, or pre-pregnancy employment. Another study found higher medication burden increased likelihood of medication-related breastfeeding discontinuation after starting olanzapine.	VERY LOW	Downgraded due to imprecision (most data coming from a single study) and possible publication bias

## Discussion

This review found that maternal medication is associated with a 2–18% likelihood of breastfeeding discontinuation in general populations. Among women with chronic diseases, this likelihood ranges from 2 to 58%. In many cases, the medicines implicated are known to be safe in breastfeeding. Healthcare professionals are likely to play a key role in influencing women’s decisions around combining medication with breastfeeding, although personal beliefs may also be important.

There is limited research exploring risk factors for medication-related breastfeeding discontinuation related to women’s personal characteristics. The certainty of evidence was rated as very low for all outcomes (Table 3), largely due to methodological weaknesses, inconsistency, and limited generalizability. These limitations reduce confidence in the effect estimates and highlight the need for higher-quality primary studies.

The findings of this review regarding factors influencing medication-related breastfeeding discontinuation are generally consistent with earlier related studies. For example, one review reported that healthcare professionals often advised against combining medication and breastfeeding, although many studies included in their review were over 20 years old [54]. They also highlighted a lack of representation from lower-income and minority ethnic groups; this limitation persists in the current review. A more recent review similarly predominantly includes papers that are over 20 years old or from lower income countries, therefore applicability may be limited. Nonetheless, similar conclusions are drawn to the present review regarding difficulties faced for breastfeeding individuals with chronic conditions, and a lack of data exploring influencing factors in depth. To our knowledge, no previous attempt has been made to identify risk factors, rather than general influencing factors, for medication-related breastfeeding discontinuation using systematic review.

Building on these earlier findings, this review reinforces the critical role that healthcare professionals play in shaping breastfeeding decisions; however inconsistencies and overly cautious advice remain a significant challenge. Multiple factors may contribute to inaccurate or overly cautious recommendations, including limited or outdated training in breastfeeding pharmacology, time constraints during clinical consultations, concerns about professional liability, and reliance on overly conservative or non-specialist information sources. In the absence of clear or accessible evidence, some professionals may err on the side of caution and advise breastfeeding cessation unnecessarily [54, 55]. Further research exploring the experiences, knowledge, and decision-making processes of different healthcare professional groups such as general practitioners, pharmacists, midwives, and lactation consultants may be invaluable in informing the development of targeted interventions and training. Universal, simple and widely publicised access to healthcare professionals specifically trained to manage medication use in breastfeeding women may begin to address some of the problems.

Women’s own concerns about combining medication with breastfeeding also influence decisions. These fears may be shaped not only by medical advice, but also by cultural beliefs, societal expectations, family dynamics and individual health literacy. For example, fear of medication harm may be compounded by social stigma around “failing” to breastfeed or a lack of visible role models combining treatment with breastfeeding. The sparse and uneven qualitative evidence currently available limits understanding of the experiential and contextual factors. There is a need for in-depth qualitative research to explore women’s perspectives, healthcare professional interactions, and the broader social and cultural contexts shaping these decisions [56]. Such work is essential to develop effective interventions, such as high-quality patient information that clearly explains the risks and benefits of medication use versus premature breastfeeding discontinuation.

This systematic review possesses several strengths, notably its comprehensive literature search and methodological rigour, which have effectively identified substantial gaps in the existing research. However, several limitations warrant consideration.

The heterogeneity and often lower quality of the included studies precluded meta-analysis and limited the ability to draw firm conclusions. While many studies were of lower methodological quality, this may reflect the practical challenges of conducting rigorous research in postpartum populations, where prospective designs may be difficult to implement and recruitment can be particularly challenging [57, 58]. Although greater consistency in study findings might have supported generalizability despite high heterogeneity, the quantitative results across studies were too variable to draw firm conclusions.

Many studies focused on specific chronic diseases rather than broader populations, restricting generalizability. Sampling biases were common, with over-representation of well-educated Caucasian women, and possible underrepresentation of those ambivalent about breastfeeding, raising equity concerns. The lack of a clear and consistent definition of medication-related breastfeeding discontinuation across studies further complicates comparisons, and, in many cases, data collection occurred before the recommended six months of exclusive breastfeeding, potentially leading to an underestimation of medication-related breastfeeding discontinuation cases. The exclusion of unpublished studies may have contributed to publication bias.

In addition to these methodological issues, biases related to participant reporting may further impact reliability of findings. Decisions and actions around breastfeeding are likely to be particularly prone to social desirability bias, yet none of the studies in this review reported strategies to mitigate it [59]. This is a notable limitation, as participants may feel pressure to report decisions they perceive as socially acceptable, such as continuing to breastfeed or following medical advice without question, rather than their true experiences or motivations. In particular, when healthcare professionals recommend discontinuing breastfeeding due to medication use, parents may underreport disagreement or alternative feeding decisions. This lack of transparency can obscure the true extent to which such recommendations influence breastfeeding discontinuation and may limit our understanding of parents’ decision-making processes in real-world contexts.

Many studies received lower scores on the Newcastle-Ottawa scale due to their reliance on written self-reports rather than structured interviews. Nonetheless, it is important to acknowledge that written reports may be advantageous in this context, as they may reduce the risk of social desirability bias.

Future research should aim to better understand the factors that influence an individual’s decision to discontinue breastfeeding due to medication use. This could be achieved by mapping these factors to an established theoretical framework, such as the Theoretical Domains Framework, to provide a clearer understanding of the underlying determinants beyond medical influences and a woman’s personal concerns such as family support, cultural norms and societal pressures [60]. Mixed-methods designs combining quantitative incidence rates with qualitative insights would be best placed to provide the detail needed to define the problem before beginning the design of interventions. The studies reviewed here primarily focused on women with specific chronic conditions. Future research should aim to include broader populations as the most commonly used drugs in postpartum women tend not to be confined to those with chronic conditions. Examples of these include vitamins, NSAIDs, paracetamol, iron, antibiotics, dermatological agents, laxatives and contraceptives [56, 61, 62]. Similarly, there are several chronic conditions poorly represented in the current literature. The most common chronic conditions found in pregnant and postpartum women include chronic lung diseases such as asthma, thyroid disorders, and mental health conditions such as anxiety and personality disorders [63]. In contrast, this review has found rheumatological conditions to be the most widely studied. High-quality studies with a diverse and representative sample of breastfeeding individuals are essential to ensure the results are generalizable. Clear and considered definitions of medication-related breastfeeding discontinuation should also be provided. Additionally, more research is needed to identify the personal characteristics that may predispose individuals to medication-related breastfeeding discontinuation.

The broader implications of this review extend beyond the immediate issue of medication-related breastfeeding discontinuation and touch on several critical areas of healthcare, public health, and societal well-being. Firstly, the findings underscore the importance of consistent, evidence-based healthcare advice to support breastfeeding, thereby improving long-term maternal and infant health outcomes and reducing healthcare costs associated with formula feeding. Supporting informed decision-making may also lessen feelings of guilt or failure, and promote equity in maternal and infant health outcomes [64–66]. The review also highlights the broader need for culturally sensitive and inclusive research practices to address health disparities. This includes the meaningful inclusion of underrepresented populations, such as women from minority ethnic backgrounds, those with lower socioeconomic status, non-native language speakers, and individuals with diverse family structures or cultural beliefs about breastfeeding. Without active efforts to engage these groups, research may continue to produce evidence and interventions that are not applicable to all, thereby reinforcing existing inequalities. Culturally inclusive research approaches, such as community-based participatory methods, tailored recruitment strategies, and culturally adapted study materials, are essential for generating findings that are relevant, equitable, and capable of informing effective policy and practice across diverse populations. Ultimately, advancing understanding of medication-related breastfeeding discontinuation and developing targeted interventions could provide a model for addressing other barriers to breastfeeding, contributing to global public health initiatives aimed at increasing breastfeeding rates.

## Conclusions

This systematic review highlights the significant role that medication use plays in breastfeeding discontinuation among postpartum women. Despite the availability of extensive resources and guidance regarding the safety of many medications during breastfeeding, many women still discontinue breastfeeding prematurely, often based on inaccurate information. The findings underscore the importance of healthcare professionals being well-informed about the safety of medications during breastfeeding to prevent unnecessary cessation.

Additionally, the review reveals significant gaps in research related to the incidence of breastfeeding discontinuation due to medication use, especially among diverse socio-economic, geographic, and health backgrounds. Understanding the factors that contribute to these decisions is essential for developing targeted interventions to support breastfeeding individuals. By addressing these gaps and improving communication between healthcare providers and breastfeeding mothers, there is potential to improve breastfeeding rates and ensure that more women and infants can experience the well-documented health benefits of breastfeeding.

Future research should prioritize longitudinal studies that explore the relationship between medication use and breastfeeding continuation in diverse populations. Addressing these gaps through further research and informed clinical practice can contribute to higher breastfeeding rates and better health outcomes for mothers and infants.

## Electronic supplementary material

Below is the link to the electronic supplementary material.

Supplementary Material 1

Supplementary Material 2

Supplementary Material 3

Supplementary Material 4

Supplementary Material 5

Supplementary Material 6

Supplementary Material 7

## Data Availability

No datasets were generated or analysed during the current study.

## Abbreviations

BMI: Body mass index
DMARD: Disease-modifying anti-rheumatic drug
GRADE: Grading of Recommendations Assessment, Development and Evaluation
IBD: Inflammatory bowel disease
NICE: National Institute for Health and Care Excellence
NSAID: Non-steroidal anti-inflammatory drug
PRISMA: Preferred Reporting Items for Systematic Reviews and Meta-Analyses
SWiM: Synthesis without meta-analysis
UK: United Kingdom
USA: United States of America
WHO: World Health Organization
