# Supplementary material for: The effect of medication use on breastfeeding continuation: a systematic review with narrative synthesis
Source: Int Breastfeed J. 2025 Aug 4;20:59. doi: 10.1186/s13006-025-00756-y (PMC12320353; doi:10.1186/s13006-025-00756-y)
Supplement: Supplementary file 1 — Supplementary Material 1 [file 13006_2025_756_MOESM1_ESM.docx]

**Appendix 1 – Search Strategy**

| Database | BREASTFEEDING | AND | MEDICATION | AND | DISCONTINUATION | Filters Applied |
| --- | --- | --- | --- | --- | --- | --- |
| Embase (Elsevier) | ‘breast feeding’/exp OR ‘breastfeeding’/exp OR ‘breastfeeding duration’/exp OR ‘breast feeding education’/exp OR ‘nursing mother’/exp OR ‘attitude to breast feeding’/exp or ‘breast feed*’:ti,ab,kw OR breastfeed*:ti,ab,kw OR ‘nursing mother*’:ti,ab,kw OR ‘nursing wom?n’:ti,ab,kw |  | ‘drug therapy’/exp OR ‘medication therapy management’/exp OR drug*:ti,ab,kw OR medication:ti,ab,kw OR prescription:ti,ab,kw OR medicin*:ti,ab,kw |  | cessation:ti,ab OR cease:ti,ab OR discontinue*:ti,ab OR stop*:ti,ab OR barrier:ti,ab OR duration: ti,ab | Publication year: 2004-2024 |
| PubMed (MEDLINE) | (breast feeding, exclusive [MeSH Terms]) OR (breastfeed*[Title/Abstract]) OR (“breast feed*”[Title/Abstract]) OR (“nursing mother*”[Title/Abstract]) OR (“nursing woman”[Title/Abstract]) OR (“nursing women”[Title/Abstract]) |  | (drug therapy[MeSH Terms]) OR (drug*[Title/Abstract]) OR (medication[Title/Abstract]) OR (prescription*[Title/Abstract]) OR (medicin*[Title/Abstract]) |  | (cessation[Title/Abstract]) OR (cease[Title/Abstract]) OR (stop*[Title/Abstract]) OR (discontinu*[Title/Abstract]) OR (barrier*[Title/Abstract]) OR (duration[Title/Abstract]) | Year 2004-2024 |
| PsycINFO (APA PsycNET) | (Index Terms: (breast feeding)) OR (title: ("breast feed*")) OR (abstract: ("breast feed*")) OR (title: ("breastfeed*")) OR (abstract: ("breastfeed*")) OR (title: ("nursing mother*")) OR (abstract: ("nursing mother*")) OR (title: ("nursing woman")) OR (abstract: ("nursing woman")) OR (title: ("nursing women")) OR (abstract: ("nursing women")) |  | (IndexTermsFilt: ("Drug Therapy") OR IndexTermsFilt: ("Chemotherapy") OR IndexTermsFilt: ("Drug Augmentation") OR IndexTermsFilt: ("Drug Administration Methods") OR IndexTermsFilt: ("Drug Dosages") OR IndexTermsFilt: ("Drug Repurposing") OR IndexTermsFilt: ("Hormone Therapy") OR IndexTermsFilt: ("Immunotherapy") OR IndexTermsFilt: ("Narcoanalysis") OR IndexTermsFilt: ("Polypharmacy") OR IndexTermsFilt: ("Prescribing (Drugs)") OR IndexTermsFilt: ("Prescription Drug Misuse") OR IndexTermsFilt: ("Prophylactic Drug Therapy") OR IndexTermsFilt: ("Psychedelic Assisted Therapy") OR IndexTermsFilt: ("Vitamin Therapy") OR IndexTermsFilt: ("Self-Medication")) OR (title: (drug*)) OR (abstract: (drug*)) OR (title: (medication)) OR (abstract: (medication)) OR (title: (prescription*)) OR (abstract: (prescription*)) OR (title: (medicin*)) OR (abstract: (medicin*)) |  | (title: (cessation)) OR (abstract: (cessation)) OR (title: (cease)) OR (abstract: (cease)) OR (title: (stop*)) OR (abstract: (stop*)) OR (title: (discontinu*)) OR (abstract: (discontinu*)) OR (title: (barrier)) OR (abstract: (barrier)) OR (title: (duration)) OR (abstract: (duration)) | Year 2004-2024 |
| The Cochrane Library and CENTRAL (Wiley Interface) | (MeSH descriptor:[Breast Feeding] explode all trees) OR ((breast NEXT feed*):ti,ab,kw) OR ((breastfeed*):ti,ab,kw) OR ((nursing NEXT mother*):ti,ab,kw) OR ((nursing NEXT wom?n):ti,ab,kw) |  | (MeSH descriptor:[Drug Therapy] explode all trees) OR ((drug*):ti,ab,kw) OR ((medication):ti,ab,kw) OR ((prescription*):ti,ab,kw) OR ((medicin*):ti,ab,kw) |  | ((cessation):ti,ab,kw) OR ((cease):ti,ab,kw) OR ((stop*):ti,ab,kw) OR ((discontin*):ti,ab,kw) OR ((barrier):ti,ab,kw) OR ((duration):ti,ab,kw) | Custom range: 01/01/2004 to 31/12/2024 |
| Scopus (Elsevier) | (TITLE-ABS-KEY(breastfeed* OR "breast feed*" OR "nursing mother*" OR "nursing wom?n")) |  | (TITLE-ABS-KEY( drug* OR medication OR prescription* OR medicin*)) |  | (TITLE-ABS-KEY (cessation OR cease OR stop* OR discontinu* OR barrier OR duration)) | Publication year: 2004 to 2024 |
| CINAHL (EBSCOHost) | (MH "Breast Feeding Promotion") OR (MH "Ineffective Breastfeeding (NANDA)") OR (MH "Breast Feeding Positions") OR (MH "Attitude to Breast Feeding") OR (MH "Latching, Breastfeeding") OR (MH "Breast Feeding") ) OR TI breastfeed* OR AB breastfeed* OR TI "breast feed*" OR AB "breast feed*" OR TI "nursing mother*" OR AB "nursing mother*" OR TI "nursing woman" OR AB "nursing woman" OR TI "nursing women" OR AB "nursing women" |  | (MH "Drug Therapy+") OR TI drug* OR AB drug* OR TI medication OR AB medication OR TI prescription* OR AB prescription* OR TI medicin* OR AB medicin* |  | TI cessation OR AB cessation OR TI cease OR AB cease OR TI stop* OR AB stop* OR TI discontinu* OR AB discontinu* OR TI barrier OR AB barrier OR TI duration OR AB duration | Published date: 01/01/2004- 31/12/2024 |
