# Supplementary material for: The effect of medication use on breastfeeding continuation: a systematic review with narrative synthesis
Source: Int Breastfeed J. 2025 Aug 4;20:59. doi: 10.1186/s13006-025-00756-y (PMC12320353; doi:10.1186/s13006-025-00756-y)
Supplement: Supplementary file 2 — Supplementary Material 2 [file 13006_2025_756_MOESM2_ESM.docx]

**Identification of studies via other methods**

**Identification of studies via databases and registers**

Records removed *before screening*:

Duplicate records removed

(n = 3060)

LactMed monographs

(n = 377)

Bioequivalence studies

(n = 101)

Date pre-2004 (n = 14)

Total (n = 3552)

Records identified from:

Citation searching (n = 1197)

Records identified from:

Embase (n = 2467)

MEDLINE (n = 1376)

Cochrane Library (n = 1068)

PsycINFO (n = 121)

CINAHL (n = 445)

Scopus (n = 2883)

Total (n = 8360)

**Identification**

Records after duplicates removed

(n = 4808)

Records excluded from title and abstract screen

(n = 4516)

Reports excluded:

Abstract only (n = 93)

Not high-income country (n = 10)

Incorrect participant population (n = 9)

Not primary research (n = 7)

Study protocol only (n = 21)

Not English language (n = 8)

Duplicate (n = 1)

Study focused on recreational drug use (n = 3)

Did not address research questions (n = 66)

Did not address medication use (n = 42)

Incorrect study design (n = 14)

Focussed on galactagogues (n = 2)

Total (n = 276)

Reports not retrieved

(n = 0)

Reports sought for retrieval

(n = 94)

Reports sought for retrieval

(n = 292)

**Screening**

Reports excluded:

Abstract only (n = 5)

Incorrect participant population (n = 3)

Not primary research (n = 1)

Did not address research questions (n = 24)

Did not address medication use (n = 52)

Incorrect study design (n = 4)

Total (n = 89)

Reports assessed for eligibility

(n = 94)

Reports assessed for eligibility

(n = 292)

Studies included in review

(n = 20)

**Included**

*From:*  Page MJ, McKenzie JE, Bossuyt PM, Boutron I, Hoffmann TC, Mulrow CD, et al. The PRISMA 2020 statement: an updated guideline for reporting systematic reviews. BMJ 2021;372:n71. doi: 10.1136/bmj.n71. For more information, visit: <http://www.prisma-statement.org/>
