# Supplementary material for: The effect of medication use on breastfeeding continuation: a systematic review with narrative synthesis
Source: Int Breastfeed J. 2025 Aug 4;20:59. doi: 10.1186/s13006-025-00756-y (PMC12320353; doi:10.1186/s13006-025-00756-y)
Supplement: Supplementary file 3 — Supplementary Material 3 [file 13006_2025_756_MOESM3_ESM.docx]

| Study and study type | Setting | Participants details | Sample size | Type of breastfeeding | Data collection and follow-up |
| --- | --- | --- | --- | --- | --- |
| De Waard et al. [34]  Prospective cohort | Netherlands  Single centre in Amsterdam | All women giving birth in obstetrics ward, or under primary midwife-led care at three associated midwifery offices.  Exclusions: life expectancy of infant less than 72 hours; unable to speak Dutch, English or French. | 292 women | Breastfeeding was defined as any means of providing the infant with mother’s own milk, whether exclusive or partial | Structured interview 2 to 5 weeks postpartum, plus data from patient’s medical chart.  Follow-up via postal questionnaire within 4 months post-delivery. |
| Gilad et al. [35]  Prospective cohort | Israel  Calls to teratology information service helpline | All breastfeeding women contacting helpline regarding the use of olanzapine | 37 women requiring olanzapine  51 women using paracetamol were employed as a control group | No detail provided | Questionnaire via telephone, one to two years following initial contact |
| Gilad et al. [36]  Prospective cohort | Israel  Calls to teratology information service helpline | Breastfeeding women contacting helpline  Exclusions: mothers not given a full course of methylergonovine; infants born with major birth defects or prematurely | 38 women requiring methylergonovine  58 women using amoxicillin were employed as a control group | No detail provided | Questionnaire via telephone, one to three years following initial contact |
| Aigner et al. [37]  Retrospective survey | Germany  Nationwide  Recruitment during hospital consultations at three hospitals, through online advertisements and via neurologists specialised in MS. | Women with multiple sclerosis who gave birth within 3 years before study commencement. | 62 women giving birth to 64 children, of whom 55 were breastfed | Results reported on exclusive breastfeeding duration in addition to total breastfeeding duration, but no detail on definitions. | Questionnaire  No follow-up |
| Baker et al. [38]  Retrospective mixed-methods survey | England and Wales  Study champions in acute psychiatric settings nationwide were used to identify eligible women. | Women with psychiatric disorder requiring acute care from at least one psychiatric service during the first year after childbirth and had capacity to consent at the point of or after discharge.  Exclusions: women under acute care prophylactically; women who had baby permanently removed prior to admission. | 218 women, of whom 144 reported initiating breastfeeding. | Women dichotomously categorized as either breastfeeding (exclusively breastfeeding, supplementing some feeds with formula milk, or giving expressed breast milk via bottle) or no breastfeeding (formula feeding only). No other detail collected. | Questionnaire at 1-month post discharge from psychiatric service. |
| Ince-Askan et al. [40]  Prospective cohort | Netherlands  Nationwide, women recruited by their rheumatologist | Women with rheumatoid arthritis who had a wish to conceive or were already pregnant  Twin pregnancies excluded | 249 pregnancies from 216 women. The paper states that in 223 pregnancies, breastfeeding was discontinued before 26 weeks. It is unclear whether breastfeeding was continued beyond 26 weeks in the remaining pregnancies or was never initiated at all. | Exclusive and partial breastfeeding combined | Questionnaire  Data collected 3 times postpartum (6, 12, and 26 weeks) |
| Kemper et al. [41]  Prospective cohort | Netherlands  Single centre | Women with rheumatoid arthritis who had given birth | 171 pregnancies; 120 women reported initiating breastfeeding | No detail provided  Breastfeeding divided into exclusive and partial.  Early discontinuation of breastfeeding was defined as stopping before 12 weeks post-partum. | Face-to-face questionnaire  Visits were scheduled during each trimester and post-partum at 4–6, 12 and 26 weeks. |
| Klevmoen et al. [42]  Retrospective survey | Netherlands and Norway  Nationwide, recruitment via clinics, patient organization mailing lists and social media | Women with familial hypercholesterolaemia who had given birth to live children  No exclusion criteria specified | 102 women, of whom 78 report initiating breastfeeding | No detail provided  Breastfeeding not defined  Mean duration of breastfeeding reported to be 7.1 months | Online questionnaire  No follow-up |
| Mills et al. [43]  Retrospective cohort | USA  Single centre | Women with autoimmune or inflammatory rheumatic diseases aged 18 to 50 who were currently pregnant or had previously had a successful pregnancy | 151 pregnancies from 90 respondents, of which 82% were breastfed. | No detail provided | Single survey. The time point of this post-partum is unclear. |
| Orefice et al. [44]  Prospective cohort | Rome  Single centre | Women with systemic lupus erythematosus, enrolled prenatally | 57 pregnancies involving 43 women. In 41 of these pregnancies breastfeeding was initiated. | Exclusive breastfeeding, complementary breastfeeding and exclusive formula were defined separately, but no detail provided in relation to breastfeeding discontinuation due to medication | Questionnaire  Women followed up at several time points both before and after birth. Information on reasons for breastfeeding discontinuation reported at 1 month only |
| Tandon et al. [45]  Prospective cohort | Canada  Single centre | Ambulatory adult women with inflammatory bowel disease presenting to a tertiary care pre- conception and pregnancy clinic | 74 women, of whom 70 initiated breastfeeding | No detail provided | Survey  Completed at delivery and postpartum at intervals of 3 months, 6 months and 12 months |
| Frayne et al. [61]  Qualitative study | Australia  Single clinic setting | Women attending an antenatal clinic for severe mental illness | 12 women | No information | Single interview at 4-6 weeks postpartum |
| Hicks et al. [62]  Cross-sectional mixed methods | USA  Recruited from a single opioid dependence treatment centre | Women over 18 who have delivered a baby while in treatment between January 2013 and May 2015 | 30 | All participants used combination feeding (breast milk and formula) | Interview  No follow-up |
| Ikram et al. [39]  Prospective cohort | USA  Single clinic | Women with rheumatic disease attending single clinic run by single consultant  No exclusion criteria specified | 265 pregnant women; 222 initiated breastfeeding | Breastfeeding defined as either exclusively or in combination with formula | Survey completed during pregnancy  Second survey completed at the postpartum visit, 5–12 weeks after delivery |
| Lewallen et al. [63]  Cross-sectional qualitative data | USA  Women recruited from community or specialty women’s hospitals in South-eastern USA during admission for childbirth | Women who gave birth to a term neonate requiring routine care only.  Inclusions: breastfeeding for the first time, intending to breastfeed for at least 8 weeks  Exclusions: under 18s; unable to speak and understand English; infant not discharged with the mother | 379 women, of which 121 had ceased breastfeeding at time of data collection | No data | Single telephone interview at 8 weeks post-partum |
| Standish et al. [49]  Qualitative | USA  Most recruitment via Boston Medical Centre, but also via other  speciality clinics and an addiction treatment facility. | English-speaking women with a diagnosis of opioid use disorder who were primary care takers of their infants. | 23 women, of whom 16 initiated breastfeeding | Women were one to seven months postpartum.  Only one participant exclusively breastfed.  Three were still breastfeeding at the time of the interview. | Semi-structured interview  Single interview only |
| Tigka et al. [48]  Prospective cohort | Greece  Recruited from five tertiary maternity hospitals in one prefecture | Mothers who had given birth and were hospitalized in the postnatal ward.  The mother-child dyad had to be in good health, the mother had to be able to communicate effectively in Greek, and have access to a permanent telephone number for follow-up | 847 women | No detail provided  Breastfeeding not defined | In-person structured interview conducted on third day postpartum.  Follow-up telephone interview at one, three, and six months postpartum |
| Zingone et al. [64]  Cross-sectional | Italy  Single centre | Women aged between 18 and 65 years; with a confirmed inflammatory bowel disease (IBD) diagnosis of at least 1 year. | 228 women with IBD, and 229 healthy controls.  61 IBD patients provide information on experience of childbirth; 41 of these breastfed | No detail provided | Single questionnaire |
| Lewkowitz et al. [46]  Randomised controlled trial | USA  Single centre | Socioeconomically disadvantaged African American women with higher BMI with established prenatal care at study centre eligible for recruitment  Inclusions: 18–35 years of age, singleton gestation, normal foetal anatomy, pre-pregnancy BMI 30-45kg/m^2^  Exclusions: history of diabetes or gestational diabetes, prior macrosomic infant, prior spontaneous preterm bit, active substance abuse with alcohol or drugs, treatment with medications known to have body weight effects (corticosteroids, antipsychotics) | 118 women; 59 assigned to each study arm | No detail provided | Women randomly assigned to Parents as Teachers (PAT)- a home-based parenting support and child development educational intervention, or PAT+, PAT with additional content on breastfeeding.  Single data collection point, 6-12 months postpartum  Telephone questionnaire |
| Teich et al. [47]  Qualitative analysis of sample of women enrolled in separate randomised controlled trial (whereby women were randomised to prenatal care with electronic prompts during visits; pre- and postnatal visits with a lactation consultant; both electronic prompts and visits from a lactation consultant; or routine prenatal care only. | USA  Participants were recruited during routine prenatal from two sites located in New York | English or Spanish speaking women over the age of 18 who were in the first or second trimester of a singleton pregnancy.  Exclusions: women with risk factors for premature birth; maternal or infant contraindications to breastfeeding | 67 women | Data on breastfeeding intensity at 1 month provided, with 19 (28.4%) being low intensity (<20%), 26 (38.8%), medium intensity (20-80%) and 17 (25.4%) being high intensity. Breastfeeding intensity was defined as the percentage of all feedings in the past 7 days that were breast milk. | Interview at 6 months post-partum  No follow-up |
