# Supplementary material for: The effect of medication use on breastfeeding continuation: a systematic review with narrative synthesis
Source: Int Breastfeed J. 2025 Aug 4;20:59. doi: 10.1186/s13006-025-00756-y (PMC12320353; doi:10.1186/s13006-025-00756-y)
Supplement: Supplementary file 4 — Supplementary Material 4 [file 13006_2025_756_MOESM4_ESM.docx]

Table 1: assessment of methodological quality of cohort studies using a modified Newcastle-Ottawa scale

*Results have been reported under the headings of selection, comparability and outcome as per the Newcastle-Ottawa scale (see Bias Assessment Tool 1 below).*

*The Newcastle-Ottawa Scale [29] was modified by the authors of this review to ensure its relevance for single cohort studies. This modification included a “not applicable” for option for questions relating to cohort comparisons.*

| Study | Selection | Comparability | Outcome | Total | Scores expressed as a percentage |
| --- | --- | --- | --- | --- | --- |
| Aigner et al. [37] | 2/3 | N/A | 1/3 | 3/6 | 50% |
| Baker et al. [38] | 3/3 | N/A | 1/3 | 4/6 | 67% |
| Gilad et al. [35] | 3/4 | 1/1 | 1/3 | 5/8 | 63% |
| Gilad et al. [36] | 3/4 | 1/1 | 1/3 | 5/8 | 63% |
| Ikram et al. [39] | 1/3 | N/A | 1/3 | 2/6 | 33% |
| Ince-Askan et al. [40] | 3/3 | N/A | 2/3 | 5/6 | 83% |
| Kemper et al. [41] | 2/4 | 0/1 | 2/3 | 4/8 | 50% |
| Lewallen et al. [63] | 3/3 | N/A | 0/3 | 3/6 | 50% |
| Mills et al. [43] | 1/3 | N/A | 0/3 | 1/6 | 17% |
| Orefice et al. [44] | 2/3 | N/A | 0/3 | 2/6 | 33% |
| Tandon et al. [45] | 1/3 | N/A | 1/3 | 2/6 | 33% |
| Tigka et al. [48] | 2/3 | N/A | 2/3 | 4/6 | 67% |

Table 2: Assessment of methodological quality of cross-sectional studies using a modified Newcastle-Ottawa scale

*Results have been reported under the headings of selection, comparability and outcome as per the Newcastle-Ottawa scale (see Bias Assessment Tool 2 below).*

*For cross-sectional studies, a Newcastle-Ottawa Scale adapted for cross-sectional studies was used [30]. This tool was further modified by the authors of this review to ensure it assessed similar areas as the original Newcastle-Ottawa Scale for cohort studies. Modifications comprised: removal of questions regarding clarity of aims and sample size, and insertion of the option to select “not applicable” for questions regarding comparability between groups to allow for studies with single outcome groups.*

| Study | Selection | Comparability | Outcome | Total | Scores expressed as a percentage |
| --- | --- | --- | --- | --- | --- |
| De Waard et al. [34] | 5/7 | 0/1 | 3/5 | 8/13 | 62% |
| Hicks et al. [62] | 2/5 | 0/1 | 3/5 | 5/11 | 46% |
| Klevmoen et al. [42] | 1/5 | 0/1 | 2/5 | 3/11 | 27% |
| Zingone et al. [64] | 2/7 | 0/2 | 3/5 | 5/14 | 36% |

Table 3: Assessment of methodological quality of randomised controlled trials studies using the Jadad scale

*Results have been reported under the headings of randomisation, blinding and dropouts as per the Jadad scale (see Bias Assessment Tool 3 below).*

| Study | Randomisation | Blinding | Dropouts | Total | Scores expressed as a percentage |
| --- | --- | --- | --- | --- | --- |
| Lewkowitz et al. [46] | 2/2 | 1/2 | 1/1 | 4/5 | 80% |

Bias assessment tool 1: modified Newcastle-Ottawa scale for cohort studies

Each star is worth one point.

**Selection**

1. Representativeness of the exposed cohort (e.g. was the study open to all breastfeeding women in an area, or only those who accessed a certain service for example).
   1. truly representative of the average breastfeeding woman/breastfeeding woman with a chronic condition in the community **🟑**
   2. somewhat representative of the average breastfeeding woman/breastfeeding woman with a chronic condition in the community **🟑**
   3. selected group of users
   4. no description of the derivation of the cohort
2. Selection of the non-exposed cohort
   1. drawn from the same community as the exposed cohort **🟑**
   2. drawn from a different source
   3. no description of the derivation of the non-exposed cohort

OR

NOT APPLICABLE as study only has a single cohort (i.e. there is no comparison with a non-exposed cohort who do not require a medication whilst breastfeeding)

1. Ascertainment of exposure
   1. secure record (eg surgical records) **🟑**
   2. structured interview **🟑**
   3. written self report
   4. no description
2. Demonstration that initiation of breastfeeding was present at start of study (e.g. was initiation of breastfeeding an inclusion criterion, or clear numbers given for the proportion who did commence breastfeeding?)
   1. yes **🟑**
   2. no/not clear

**Comparability**

1. Comparability of cohorts on the basis of the design or analysis
   1. study controls for _____________ (select the most important factor) **🟑**
   2. study controls for any additional factor **🟑** (This criteria could be modified to indicate specific control for a second important factor.)

OR

NOT APPLICABLE as there is only a single cohort (i.e. there is no comparison with a non-exposed cohort who do not require a medication whilst breastfeeding)

**Outcome**

1. Assessment of outcome
   1. independent blind assessment **🟑**
   2. record linkage **🟑**
   3. self-report
   4. no description
2. Was follow-up long enough for outcomes to occur
   1. yes (>6 months) **🟑**
   2. no/not clear
3. Adequacy of follow up of cohorts
   1. complete follow up - all subjects accounted for **🟑**
   2. subjects lost to follow up unlikely to introduce bias - small number lost - > 80 % follow up, or description provided of those lost) **🟑**
   3. follow up rate < 80% and no description of those lost
   4. no statement

Bias assessment tool 2: modified Newcastle-Ottawa scale for cross-sectional studies

Each star is worth one point.

**Selection**

1. Representativeness of the sample (e.g. was the study open to all breastfeeding women in an area, or only those who accessed a certain service for example).
   1. truly representative of the average breastfeeding woman/breastfeeding woman with a chronic condition in the community (including if only one institution)**🟑🟑**
   2. somewhat representative of the average breastfeeding woman/breastfeeding woman with a chronic condition in the community (e.g. non-random sampling)**🟑**
   3. selected group of users
   4. no description of the derivation of the cohort
2. Selection of different groups
   1. drawn from the same community as the exposed cohort **🟑🟑**
   2. drawn from a different source **🟑**
   3. no description of the derivation of the non-exposed cohort

OR

NOT APPLICABLE as study only has a single group

1. Non-respondents
   1. Response rate is assessed, is, satisfactory (>70%), and comparability between respondent and non-respondent characteristics is assessed**🟑🟑**
   2. Two of the three above is present**🟑**
   3. One or none of the above is present
2. Ascertainment of exposure (to medication while breastfeeding)
   1. secure record (eg medical records) **🟑**
   2. structured interview 
   3. written self report
   4. no description

**Comparability**

1. Confounding factors are controlled.
   1. The study controls for potential confounder(s)**🟑**
   2. No control for potential confounders
2. The subjects in different outcome groups are comparable, based on study design or analysis
   1. Yes **🟑**
   2. No

OR

NOT APPLICABLE because there was a single outcome group only

**Outcome**

1. Assessment of outcome
   1. independent blind assessment **🟑🟑**
   2. record linkage **🟑🟑**
   3. self-report **🟑**
   4. no description
2. Was the time point of data collection postpartum appropriate?
   1. Yes- at least 6 months postpartum **🟑**
   2. No- less than six months postpartum
3. Statistical testing
   1. The statistical test used to analyse the data is clearly described and appropriate, and the measurement of association is presented, including confidence intervals and p value **🟑🟑**
   2. Two of the three items above are present **🟑**
   3. The statistical test is not appropriate, not described, or incomplete

Bias assessment tool 3: Jadad scale

Each of the following criteria are scored, and points are awarded as follows:

- Randomization: 1 point if the study is described as randomized, with an additional point if the randomization method is appropriate.
- Blinding: 1 point if the study is described as double-blind, with an additional point if the method of blinding is appropriate.
- Withdrawals and dropouts: 1 point if the study provides a description of withdrawals and dropouts.

Scores range from 0 to 5, with higher scores indicating better methodological quality.
