# Supplementary material for: The effect of medication use on breastfeeding continuation: a systematic review with narrative synthesis
Source: Int Breastfeed J. 2025 Aug 4;20:59. doi: 10.1186/s13006-025-00756-y (PMC12320353; doi:10.1186/s13006-025-00756-y)
Supplement: Supplementary file 5 — Supplementary Material 5 [file 13006_2025_756_MOESM5_ESM.docx]

*Supplementary* *table 1: medicines contributing to women discontinuing breastfeeding*

| **Study and study type** | **Results**  ***Where available, n = number of women discontinuing due to the drug/drug class*** | **Breastfeeding compatibility information**  ***Data taken from Hale’s Medications and Mothers’ Milk unless otherwise stated [18]*** |
| --- | --- | --- |
| Aigner et al. [37] | Immunotherapy (n = 16)  No elaboration on which immunotherapies this concerned | Common immunotherapies used in MS:  Limited data, probably compatible (L2);   - Dimethyl fumarate - Interferon beta   Limited or no data, probably compatible (L3):   - Glatiramer - Ocrelizumab - Natalizumab   No data, possibly hazardous (L4):   - Alemtuzumab   No data, hazardous (L5):   - Fingolimod |
| Baker et al. [38] | Atypical antipsychotics such as quetiapine and olanzapine  SSRIs such as sertraline, citalopram and fluoxetine  Lithium (n = 1)  No other quantitative detail provided | Limited data, probably compatible (L2):   - Quetiapine - Olanzapine - Sertraline - Citalopram - Fluoxetine   Limited data, possible hazardous (L4):   - Lithium |
| Frayne et al. [61] | Lithium (n =1) | Lithium: limited data, possibly hazardous (L4) |
| Gilad et al. [35] | Olanzapine (n = 4)  Paracetamol (n = 0) | Olanzapine: limited data, probably compatible (L2)  Paracetamol: extensive data, compatible (L1) |
| Gilad et al. [36] | Methylergonovine (n = 7)  Amoxicillin (n = 3) | Methylergonovine: limited data, probably compatible (L2)  Amoxicillin: limited data, compatible (L1) |
| Hicks et al. [62] | Methadone  Note: this paper was excluded from the primary research question as methadone was not newly initiated during the breastfeeding period. However, four women (16%) reported long-term methadone use being the reason for stopping breastfeeding early. | Methadone: significant data, compatible (L2) |
| Ince-Askan et al. [40] | Of the 129 women who discontinued breastfeeding due to needing to restart medication, 76 cases (59%) involved a medication considered incompatible with breastfeeding. The remaining 53 cases (41%) were due to a combination of: prednisone (n = 26), sulfasalazine (n=21), hydroxychloroquine (n=10), and/or nonselective NSAID (n=30), all considered safe during breastfeeding.  No detail provided on what the incompatible medicines are. | Prednisone: limited data, probably compatible (L2)  Sulfasalazine: limited data, probably compatible (L3)  Hydroxychloroquine: limited data, probably compatible (L2)  Non-selective NSAIDs:   - Ibuprofen: extensive data, compatible (L1) - Naproxen: limited data, probably compatible (L3) - Diclofenac: limited data, probably compatible (L2)   Note: current guidance from the American College of Rheumatology, the British Society for Rheumatology and European Alliance of Associations for Rheumatology all agree that most medications used to manage rheumatic diseases are compatible with breast feeding, including antimalarials, sulfasalazine, azathioprine, ciclosporin, tacrolimus, colchicine, intravenous immunoglobulin, steroids and biologics.  Incompatible medication includes cyclophosphamide, mycophenolate mofetil, methotrexate, leflunomide and tofacitinib [36]. |
| Kemper et al. [41] | Prednisone was the drug implicated in one case of medication related breastfeeding discontinuation (7%)  No other detail is provided on the agents causing breastfeeding discontinuation in the remaining women. | Prednisone: limited data, probably compatible (L2) |
| Klevmoen et al. [42] | Statins (n = 17)  No detail on which statins are concerned | For all four available statins: no data, probably compatible (L3) |
| Lewallen et al. [63] | Paper reports examples of medications causing breastfeeding discontinuation include antibiotics and oral contraceptives. 15 of 121 (12%) women cite medication as being the reason for breastfeeding discontinuation, but no other quantitative detail is provided. | The use of antibiotics does not generally necessitate suspension or cessation of breastfeeding (65).  Progesterone-only contraceptives can be used when breastfeeding from 3 weeks after delivery. Latest guidance states combined oral contraceptives can safely be used in breastfeeding from 6 weeks postpartum. However, older guidance advised their avoidance within the first 6 months (66). |
| Mills et al. [43] | No detail provided on exactly which medicines are implicated. Medicines documented as being used by cohort are: hydroxychloroquine, azathioprine, mycophenolate mofetil, sulfasalazine, methotrexate, leflunomide, ciclosporin, prednisolone, NSAIDs, etanercept, infliximab, adalimumab, certolizumab pegol, golimumab, rituximab, narcotics and paracetamol. | See under Ince-Askan et al. (39); most medicines used in rheumatic conditions are considered safe for use during breastfeeding. Unsafe medication include cyclophosphamide, mycophenolate mofetil, methotrexate, leflunomide and tofacitinib. |
| Orefice et al. [44] | Azathioprine (n=1) | Limited data, probably compatible (L3) |
| Standish et al. [49] | Buprenorphine/naloxone (n=1) | No data, probably compatible (L3) |
| Tigka et al. [50] | Among mothers who decided to discontinue breastfeeding based on their own decision (n=7):   - Escitalopram (n=3) - Methylprednisolone (n=1) - Methyldopa and nifedipine (n=1) - Doxycycline and piperacillin-tazobactam (n=1) - Clindamycin (n=1)   Among women who discontinued breastfeeding due to a reduction in milk supply resulting from a physician’s advice to discard milk or reduce breastfeeding frequency (n=6):   - Topical gentamicin-dexamethasone - Methyldopa - Co-amoxiclav - Diclofenac - Metronidazole - Ciprofloxacin - Clindamycin   Among women who discontinued breastfeeding based on physician recommendations (n=42):   - 5 (12%) used medicines classified as possibly hazardous or hazardous - 21 (50%) used medicines identified as judicious, not classified or to be used with caution - 15 (36%) used medicines compatible with breastfeeding.   One woman was hospitalized and could not recall medication given but was advised to discontinue breastfeeding.  No further detail on medications provided | Escitalopram: L2- limited data, probably compatible  Methylprednisolone: L2- limited data, probably compatible  Methyldopa: L2- limited data, probably compatible  Nifedipine: L2- limited data, probably compatible  Doxycycline: L3- limited data, probably compatible  Piperacillin-tazobactam: L2- no data, probably compatible  Clindamycin: L2- limited data, probably compatible  Topical gentamicin-dexamethasone:   - For systematic gentamicin: L2-limited data, probably compatible - For systemic dexamethasone: L3- no data, probably compatible   (Exposure to infant via breastmilk through topical use is likely minimal)  Co-amoxiclav: L1- extensive data, compatible  Diclofenac: L2- limited data, probably compatible  Metronidazole: L2- limited data, probably compatible  Ciprofloxacin: L3- limited data, probably compatible |
| Zingone et al. [64] | Mesalamine | Mesalamine: limited data, probably compatible (L3) |

*Supplementary* *table 2: factors influencing a woman in choosing to discontinue breastfeeding due to the concurrent use of medication*

| **Study and study type** | **Results** |  |
| --- | --- | --- |
| **Studies highlighting the role of healthcare professionals** | |  |
| Baker et al. [38] | 57 women (26%) received advice to stop breastfeeding by healthcare professionals, due to the use of medication. This includes acute psychiatry services (40%, n = 22), maternity services (32.7%, n = 18) and postnatal universal services, such as health visitors, community midwives and GPs (27.3%, n=15). 43 of these women (75%) discontinued breastfeeding because of this advice.    Free-text responses identified themes of “unsupportive and inconsistent” advice regarding medication, and “de-prioritization of breastfeeding intentions”. |  |
| Kemper et al. [41] | Medication related breastfeeding discontinuation was reported less often in the study compared with the historical reference cohort (19% vs 58%). Only one patient stopped breastfeeding due to a compatible medication. This compares with 42% In the historical reference cohort. Study suggests this highlights the importance of counselling on the use of DMARDS post-partum. |  |
| Lewkowitz et al. [46] | On a one to four scale, with four denoting ‘‘very important,’’ women in PAT+ and PAT rated medication being a reason for breastfeeding discontinuation as 1.40 and 1.86 respectively (RR = 0.52, 95% CI 0.23-1.1) |  |
| Orefice et al. [44] | The one patient who reported breastfeeding discontinuation due to medication states that this was on the recommendation of a paediatrician. |  |
| Teich et al. [47] | Paper reports women in the groups without a lactation consultant were less likely to overcome medication-related barriers, although no quantitative data was provided.  Paper states several women in the lactation consultant intervention groups, who had unexpected hospital courses or needed medications, recalled working with the lactation consultants to clarify medication safety and management of breastfeeding. |  |
| Tigka et al. [48] | Of the women reporting medication-related breastfeeding discontinuation:   - 42 (74%) based their decision on the counselling of a physician (either an obstetrician or the medical specialist responsible for the treatment of the chronic disease or acute incident) - Six (11%) were advised to discard breast milk or reduce breastfeeding frequency while using a medication. One of these women decided to cease breastfeeding immediately; the remaining five followed the physician’s advice. All five of these women reported that this led to cessation of breastfeeding due to a reduction in milk supply. |  |
| **Studies highlighting personal views of women** | |  |
| Gilad et al. [35] | Four women (11% of total sample) did not breastfeed due to fear of olanzapine effects on the infant.  11 women stopped breastfeeding due to “medical advice”. No further explanation is provided, and whether this concerns medication. One woman also stopped breastfeeding due to fear. It is unclear if this is related to medication. | |
| Gilad et al. [36] | Of the 38 women for whom follow-up was obtained, six stopped breastfeeding immediately because of “concerns regarding methylergonovine treatment”. No further elaboration provided.  An additional study mother and three control mothers later discontinued breastfeeding due to concerns with possible infant drug exposure. No further elaboration provided. | |
| Standish et al. [49] | Scepticism and Concerns about Medication Exposure:  “*I thought that taking Suboxone and giving her the breast, it was like it was hurting her feeding. The doctor would tell me no, that there was no issue with that, but I felt deep down inside that there was, so I stopped [breastfeeding].*  Perceived Control Over Breastfeeding Decisions:  In some situations, mothers did not have control over their breastfeeding decisions. Some reported being told by hospital providers that they could not breastfeed due to maternal medications, smoking, or reasons unclear or unknown to the mother. ‘‘*He did actually [breast]- feed one time. But then [the hospital providers] didn’t allow me to continue and why that is? I’m not sure’’*  Beliefs about the impact of breastfeeding on infant withdrawal:  *“At some point he would still have to come off of [the Suboxone] because of the Suboxone [in the milk]. At what point do I stop breastfeeding for him to get off the Suboxone?. So, it felt like let’s just get this over with immediately. if he was going to withdraw or anything like that, we could just get that all the way, then move on with our lives.’’* | |
| Tigka et al. [48] | Of the women reporting medication-related breastfeeding discontinuation, nine (16%) based their decision on fears of harming their infant. | |

*Supplementary table 3: studies reporting on the association between a woman’s background characteristics and the likelihood of medication-related breastfeeding discontinuation*

| **Study and study type** | **Results** |
| --- | --- |
| Gilad et al. [35] | The maternal characteristics were similar in most respects; however, multiple drug therapy was more common among women who used olanzapine but decided not to breastfeed. |
| Tigka et al. [48] | Univariate logistic regression analysis found the following factors to be positively associated with medication-related breastfeeding discontinuation:   - Lower educational level (p=0.034) - Birth via Caesarean section (p=0.001) - Use of medication for chronic condition (p<0.001) - Employment at 6 months postpartum (p<0.001) - Fewer days breastfeeding experience (p<0.001) - Physician recommendation to discontinue breastfeeding due to medication (p<0.001)   When multiple logistic regression analysis was applied, smoking before pregnancy was also found to be associated with medication-related breastfeeding discontinuation (p<0.001).  No association was found between medication-related breastfeeding cessation and the following variables:   - Maternal age - Nationality - Parity - Employment before pregnancy |
